# Rapid Internalization of the Oncogenic K^**+**^ Channel K_V_10.1

**DOI:** 10.1371/journal.pone.0026329

**Published:** 2011-10-12

**Authors:** Tobias Kohl, Eva Lörinczi, Luis A. Pardo, Walter Stühmer

**Affiliations:** 1 Max-Planck-Institute of Experimental Medicine, Department of Molecular Biology of Neuronal Signals, Göttingen, Germany; 2 DFG Research Center for Molecular Physiology of the Brain (CMPB), Göttingen, Germany; Sackler Medical School, Tel Aviv University, Israel

## Abstract

K_V_10.1 is a mammalian brain voltage-gated potassium channel whose ectopic expression outside of the brain has been proven relevant for tumor biology. Promotion of cancer cell proliferation by K_V_10.1 depends largely on ion flow, but some oncogenic properties remain in the absence of ion permeation. Additionally, K_V_10.1 surface populations are small compared to large intracellular pools. Control of protein turnover within cells is key to both cellular plasticity and homeostasis, and therefore we set out to analyze how endocytic trafficking participates in controlling K_V_10.1 intracellular distribution and life cycle. To follow plasma membrane K_V_10.1 selectively, we generated a modified channel of displaying an extracellular affinity tag for surface labeling by α-bungarotoxin. This modification only minimally affected K_V_10.1 electrophysiological properties. Using a combination of microscopy and biochemistry techniques, we show that K_V_10.1 is constitutively internalized involving at least two distinct pathways of endocytosis and mainly sorted to lysosomes. This occurs at a relatively fast rate. Simultaneously, recycling seems to contribute to maintain basal K_V_10.1 surface levels. Brief K_V_10.1 surface half-life and rapid lysosomal targeting is a relevant factor to be taken into account for potential drug delivery and targeting strategies directed against K_V_10.1 on tumor cells.

## Introduction

Protein turnover within cells plays a key role in maintaining cellular homeostasis and plasticity. Here we report an analysis of the mechanisms controlling the surface expression and turnover of the oncogenic voltage-gated K^+^ channel K_V_10.1.

K_V_10.1 (Eag1) is a voltage-gated, delayed rectifier K^+^ channel from the ‘*Ether-à-go-go*’ (*KCNH*) gene family [1], [2]. It is mainly found in distinct neuronal tissues at both the mRNA and protein level [3], [4], [5]. Yet K_V_10.1 is overexpressed in a wide range of solid tumors [4]. In this context K_V_10.1 is emerging as a prognostic marker for poor outcome and as a drug-target for K_V_10.1-positive tumors [4], .

The precise mechanism how K_V_10.1 promotes proliferation of cancer cells is still under debate, although it is known that it includes both permeation-dependent and -independent components. Notably, non-conducting signaling functions might rely on ion channel conformation [9], [10], [11]. Ectopic expression of K_V_10.1 at the cell surface has been proven relevant for tumor biology, since a K_V_10.1-specific blocking antibody reduces proliferation in a variety of cancer cell types expressing K_V_10.1 both *in vitro* and *in vivo* [12]. Furthermore, experimental evidence supports a role for K_V_10.1-mediated currents in facilitating cell-cycle progression: progression through the early G1 phase of the cell cycle is promoted by membrane hyperpolarization [13], and K_V_10.1 -mediated K^+^ efflux could contribute to this hyperpolarization, a model that is also confirmed by the anti-proliferative effects of K_V_10.1 channel blockers [10], [12], [14], [15], [16], [17]. K_V_10.1 was also shown to be a cell-cycle regulated channel: K_V_10.1 currents are down-regulated at the G2-M transition, upon cell differentiation and also within cells arrested in G_0_/G_1_ [14], [18], [19]. It is likely that these events are regulated both at the level of channel activity and surface expression. In neurons, the surface-expression of endogenous K_V_10.1 is tightly controlled. No currents mediated by endogenous K_V_10.1 in neuronal tissue have been published to date in spite of the fact that in *Drosophila, eag* modulates K^+^ currents and synaptic function [20], [21], [22], [23], [24]. We recently identified a small K_V_10.1 surface population localizing preferentially to presynaptic membranes in rat hippocampal neurons, while large intracellular pools of K_V_10.1 can be readily detected in permeabilized cells [25], [26]. K_V_10.1 channels activate at sub-threshold potentials and show progressively slower activation kinetics at hyperpolarized prepulse potentials, a feature reminiscent of the Cole-Moore shift described on squid axon channels [27], [28]. These properties fit well a role in modulating both membrane resting potentials and excitability.

So far the mechanisms controlling surface expression and down-regulation of K_V_10.1 are largely unknown, but recent findings suggest that K_V_10.1 at the cell surface is rapidly turned over. Interestingly, epsin has been reported to interact with K_V_10.1 and modulate its gating in rat brain [29]. Since epsin is involved in clathrin-mediated endocytosis (CME), this interaction suggests a role for the endocytic machinery in controlling K_V_10.1 channels at the plasma membrane [30]. Silencing K_V_10.1 expression in cancer cells with siRNA treatment revealed that K_V_10.1 has a turnover of 8–12h [31]. Most likely this reflects continuous removal of K_V_10.1 from the plasma membrane by rapid endocytosis, followed by its transport to lysosomes.

In general, endocytosis contributes to the control of ion channel surface expression in neurons and epithelial cells, possibly being part of the constitutive cycling of transmembrane proteins that has been suggested to be a general mechanism in the regulation of cell surface molecules [32], [33]. Alternative mechanisms include the control and limitation of surface expression, including channel assembly and retention in the ER, post-translational modifications in the trans-Golgi network, endocytosis and related sorting processes [34], [35]. So far expression of K_V_10.1-mediated currents has been found to depend on proper channel assembly and channel glycosylation in the ER and trans-Golgi-network, respectively [36], [37].

Here we report the analysis of the K_V_10.1 life cycle with respect to endocytosis and intracellular sorting. Our report is based on a modified version of this channel displaying an extracellular affinity tag for surface labeling. Using a combination of microscopical and biochemical techniques we show that K_V_10.1 is constitutively internalized involving clathrin-mediated endocytosis and rapid sorting to lysosomes. Obviously the design of drug-delivery and targeting strategies directed against K_V_10.1 at the surface of tumor cells needs to be adapted to the surface half-life of K_V_10.1 and might also exploit its internalization [38], [39].

## Results

### Insertion of the bungarotoxin-binding site (BBS) into an extracellular loop of K_V_10.1 allows for specific surface-labeling

**Figure 1:**
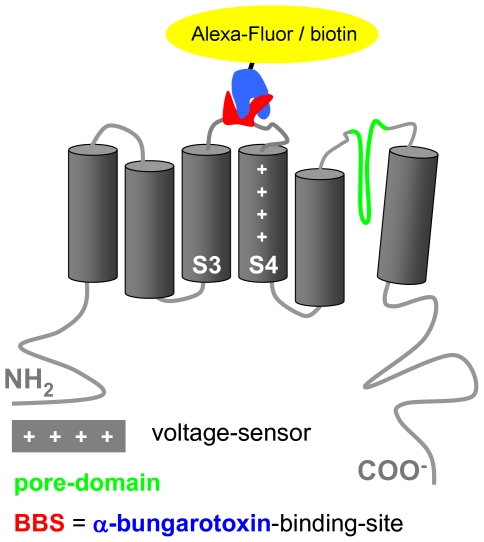
K_V_10.1-BBS is a tagged version of K_V_10.1. K_V_10.1-BBS is a voltage-gated ion channel which contains an extracellular loop with the α-bungarotoxin-binding site (BBS) that can bind α-bungarotoxin-conjugates (BTX-XX).

Previous efforts to achieve surface labeling of K_V_10.1 on living cells with antibody-based or chemical approaches resulted in modest labeling even for over-expressed K_V_10.1. Consequently, we introduced an affinity tag into the second extracellular loop of the channel mimicking the size and site of insertion of additional 27aa observed in the long K_V_10.1 splice variant [40]. The binding site for α-bungarotoxin (BTX) from the acetylcholine receptor binds BTX and its conjugates with a K_d_ in the low nanomolar range [41], [42], [43], [44]. We inserted the BTX binding site (BBS) into K_V_10.1 to generate a construct named K_V_10.1-BBS [43] (Fig. 1).

**Figure 2:**
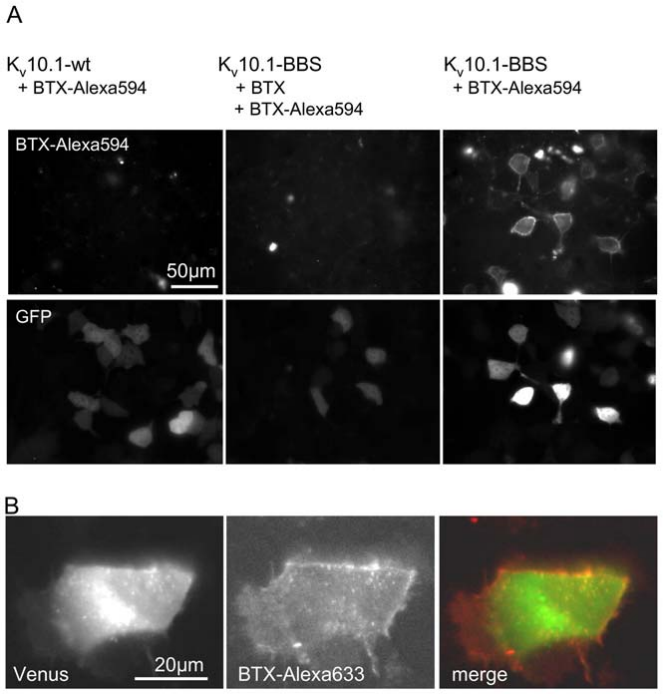
Labeling of K_V_10.1-BBS with fluorescent BTX conjugates specifically labels the cell membrane. A) Incubation of cells with BTX-Alexa594 results in membrane stains (top row, right)) in Hek cells transfected with K_V_10.1-BBS. This labeling is blocked by preincubation of cells with unlabeled BTX (center). No labeling is detectable in cells expressing wild type K_V_10.1 (left). Transfected cells can be identified based on expression of GFP from the pTracer plasmid (bottom). GFP signals do not correlate to K_V_10.1-BBS expression levels. B) Double-labeling K_V_10.1: Cells expressing the fusion protein K_V_10.1-BBS-Venus were labeled with BTX-Alexa647 to distinguish the membrane versus internal population of K_V_10.1.

At first we confirmed that surface labeling of K_V_10.1-BBS channels is specific. Surface labeling was analyzed after incubating cells expressing either K_V_10.1-BBS or wild type K_V_10.1 or mock-transfected cells with excess amounts of fluorescent BTX conjugate (2 µM) for 10 min on ice. To improve labeling efficiency on ice for short pulses, we applied ligand concentrations of two to three orders of magnitude above the K_d_ of the BBS. Only surface labeling specific for K_V_10.1-BBS could be observed (Fig. 2A). Labeling was blocked by preincubation of K_V_10.1-BBS expressing cells with 5 µM unlabeled BTX, indicating specific binding.

Next we compared the cell distribution of K_V_10.1-BBS using K_V_10.1-BBS-Venus, a C-terminal fusion of the yellow fluorescent protein Venus to K_V_10.1-BBS. Venus fluorescence typically was ubiquitous throughout the cell and showed high perinuclear intensity (Fig. 2B). In contrast, labeling with 0.3 µM BTX-Alexa633 for 10 minutes at 37°C resulted in a surface stain, as shown in Fig. 2A.

### Insertion of BBS into K_V_10.1 and BTX binding render functional channels

**Figure 3:**
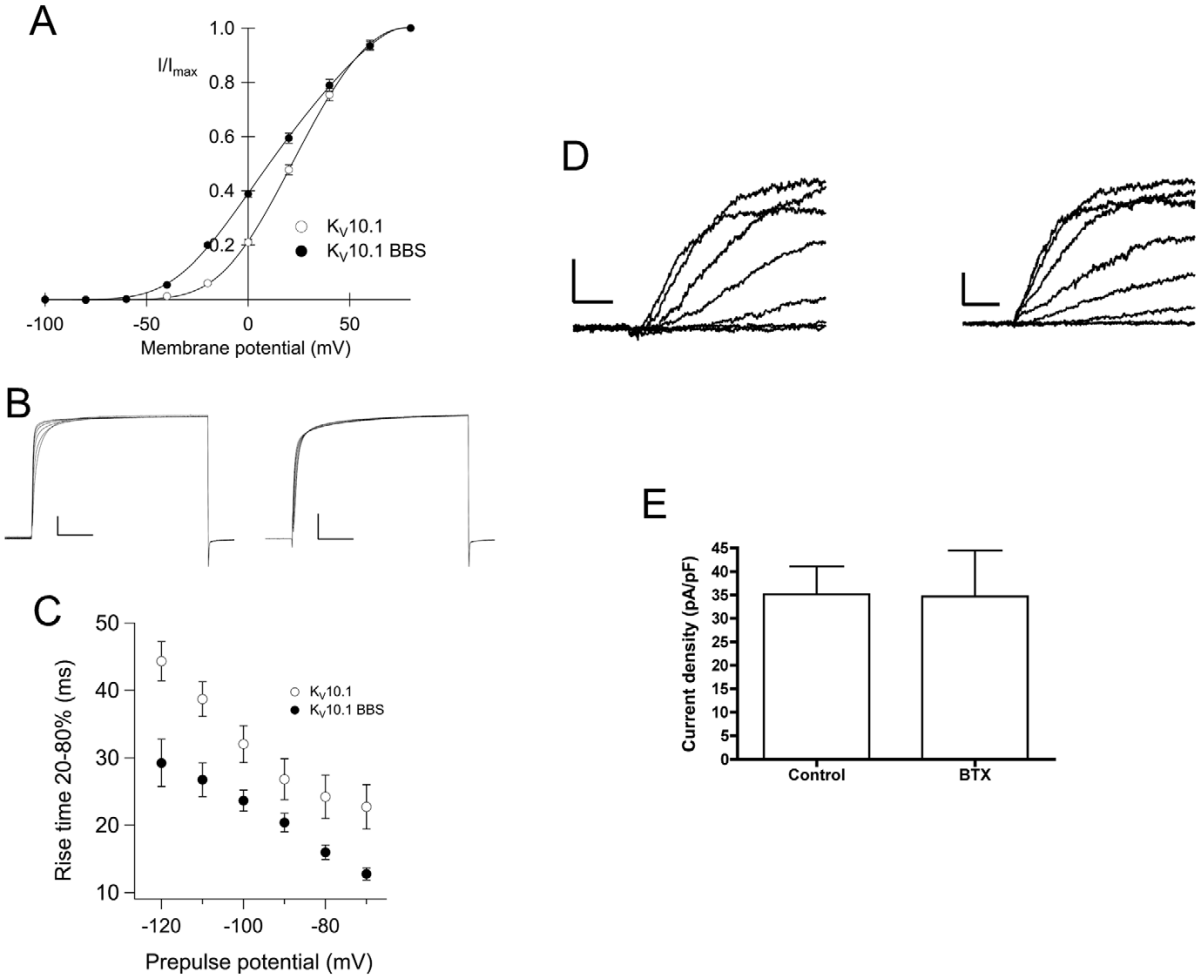
K_V_10.1-BBS with or without bound BTX-conjugates shows electrophysiological behavior similar to K_V_10.1. K_V_10.1-BBS with or without bound BTX-conjugates shows electrophysiological behavior similar to Kv10.1. A) The K_V_10.1-BBS current-voltage relationship is shifted to more negative values. Whole cell currents were triggered by stepping from a holding potential of −100 mV to test potentials (−100 mV to +80 mV) for 500 ms. Current amplitudes at the end of test pulses were normalized to amplitudes recorded at +80 mV (I/Imax) and plotted against the applied membrane potential. K_V_10.1-BBS (filled circles, n = 11) activates at more negative potentials compared to K_V_10.1 (open circles, n = 28). B) K_V_10.1 and K_V_10.1-BBS channel activation depends on the holding potential. Current traces were measured upon application of 500 ms depolarization to +40 mV after conditioning pulses (5000 ms) at potentials ranging from −120 mV to −70 mV in 10 mV increments. (C) The rise time of activation from 20 to 80% of maximal current was plotted against the holding potential. K_V_10.1-BBS (filled circles, n = 11) is characterized by shorter rise time (faster activation) as compared to K_V_10.1 (open circles, n = 28). D) Representative traces of Kv10.1-BBS mediated currents in unlabeled (left; scale bars, 1 nA, 50 ms) and labeled (right; scale bars, 0.25 nA, 50 ms) cells. No changes in kinetics were observed. E) Current density of K_V_10.1-BBS cells did not change upon binding of BTX-Alexa488 to the BBS-site.

In order to assess the effects of the BBS-tag on the function of the wild-type channel, we expressed the K_V_10.1-BBS construct in *Xenopus* oocytes. The measured currents strongly resembled those of K_V_10.1 (Fig. 3).

The long K_V_10.1 splice variant (K_V_10.1b) identified in the bovine retina [40] activates at more negative potentials than K_V_10.1a. The current-voltage relationship of K_V_10.1-BBS was also shifted to more negative membrane potentials (Fig. 3A). The half-activation potential shifted from −26 in K_V_10.1 to −54 mV in K_V_10.1-BBS. The voltage dependence of both constructs was almost identical with a slope of 26.23±4.24 mV for K_V_10.1 and 25.63±1.76 mV for K_V_10.1-BBS. Also, both constructs displayed rectification at very positive potentials [45].

The activity of KV10.1-BBS was strongly dependent on the membrane holding potential, the hallmark property of KV10.1 (Fig. 3B,C) [27]. The activation of KV10.1-BBS was faster compared to the untagged KV10.1 over the measured range of -120 to -70 mV prepulses. These data suggest that the inserted 27 amino-acid residues rendered a functional channel in in the oocyte system that resembles the properties of the longer KV10.1 splice variant.

Next, we tested if labeling K_V_10.1-BBS with BTX conjugates affects channel gating. For this purpose we analyzed currents of labeled and unlabeled cells expressing K_V_10.1-BBS. Labeling with BTX-Alexa488 was confirmed prior to electrophysiological recordings by visual inspection. Binding of BTX-Alexa488 did not alter K_V_10.1-BBS mediated K+ currents nor affected K_V_10.1-BBS dependent current densities in stably transfected HEK cells (Fig. 3D and E). The activation of BTX-labeled channels maintained the typical dependence on prepulse potential described above for the current expressed in oocytes.

### Surface K_V_10.1-BBS shows fast turnover

**Figure 4:**
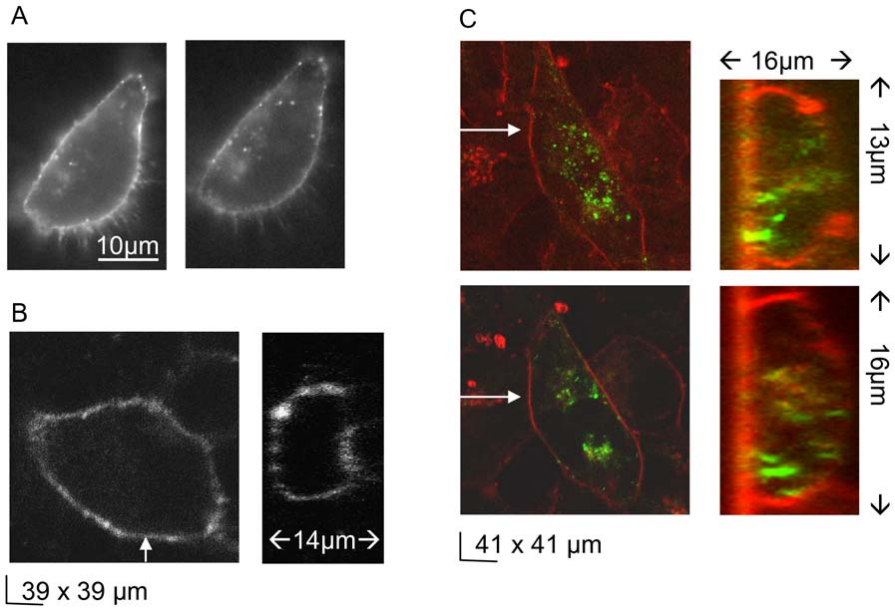
K_V_10.1 in the plasma membrane is rapidly transported to punctuate endosomal stuctures. A) Formation of vesicular structures proceeds immediately after surface -labeling of K_V_10.1-BBS with BTX-Alexa594 during 5 minutes at 30°C (left to right). Confocal laser scans were performed B) after surface-labeling K_V_10.1-BBS with BTX-Alexa488 and C) after 30 minutes of incubation at 30°C. Intracellular vesicles were identified in xy-sections and xz-projections at positions indicated by arrows (green and gray: BTX-Alexa488, red: membrane stain with FM 4–64). More endosomal structures appear in the plane of the basal membrane than 3 µm above (C, top and bottom row, respectively).

During examination of fluorescent surface stains, we observed rapid formation of punctuate patterns; we therefore hypothesized that K_V_10.1-BBS shows rapid internalization. When cells were labeled on ice and subsequently observed in a heated microscope stage at 37°C, formation of punctuate structures proceeded within 5 minutes (Fig. 4A). To better characterize this phenomenon, cells were labeled with BTX-Alexa488 and incubated at either 4°C (Fig. 4B) or 37°C (Fig. 4C) for 30 minutes. Afterwards the plasma membrane was labeled with the membrane dye FM 4–64 in order to discriminate BTX-Alexa488 surface signals more clearly from internalized signals (Fig. 4C). xz-projections of consecutive confocal sections confirmed that the punctuate signals in the green detection channel are compatible with internalized vesicles. We observed the highest number of vesicles in confocal sections acquired near the membrane located above the fibronectin-coated coverslip.

**Figure 5:**
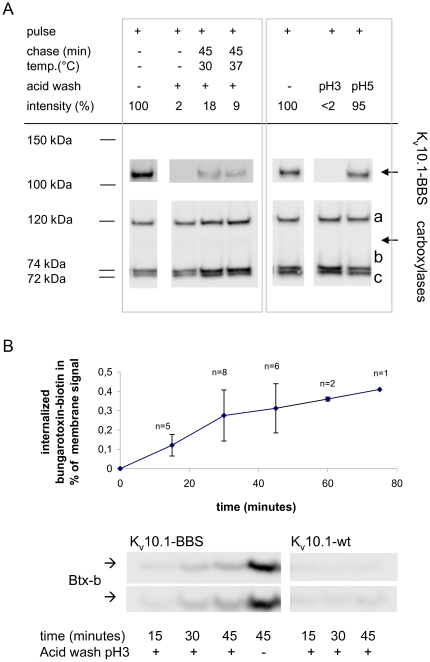
Endocytosis of K_V_10.1 is constitutive and shows saturation after 45 minutes. A) left) Internalized K_V_10.1-BBS molecules were detected in western blots (row 1, lane 3 & 4) and correspond up to ∼20% of initially labeled surface-channels (lane 1). Intracellular K_V_10.1-BBS molecules were discriminated from surface molecules by removing surface-labels using acid wash before harvest and pull-down. Endogenous biotinylated carboxylases were detected with streptavidin-peroxidase in western blots to correct for slight variations in pull-down and blotting efficiency. (*From top to bottom: a:* pyruvate-carboxylase, b: propionyl-CoA carboxylase, c: methycrotonyl-CoA carboxylase [46], [47]). Right: Acid washing at pH3 removes surface-labels while washing at pH5 does not. B) The endocytosis rate of K_V_10.1 was measured by determining the cellular uptake of BTX-biotin via K_V_10.1-BBS surface-molecules. The relative amount of internalized BTX-biotin was plotted over time (error bars: SD; top) and starts to saturate after 45 minutes. To generate this data, internalized BTX-biotin was blotted on membranes and detected with streptavidin-peroxidase (bottom, representative blot of duplicates). Ratios were determined as ‘intracellular signal/(whole-cell signal – intracellular signal)’ and corrected for unspecific uptake of BTX-biotin in cells expressing K_V_10.1. Whole-cell signals (lane 4) were determined after omitting acid washing.

We alternatively confirmed K_V_10.1-BBS internalization by detecting both surface and internalized channels directly with K_V_10.1 antibody in western blots (Fig.5A). For this purpose HEK cells stably transfected with K_V_10.1-BBS were labeled with BTX-biotin on ice for 10 minutes (pulse) and incubated in growth medium for 45 minutes at 30°C or 37°C (chase). After the chase period, the remaining surface-label was removed by acid wash at pH3. Cells were then lysed and internalized K_V_10.1-BBS bound to BTX-biotin was pulled-down from lysates using magnetic streptavidin beads. Internalized K_V_10.1-BBS corresponded to 20% of the initial surface labeling. The interaction of BTX-biotin and K_V_10.1-BBS survived acid washing at pH5, but not pH3 of adherent labeled HEK cells for 2 minutes at 8°C. We noticed that endogenously biotinylated mitochondrial carboxylases are pulled-down along with biotin-labeled K_V_10.1-BBS channels [46], [47]. These endogenously biotinylated proteins provided an internal control for both pull-down and blotting efficiency.

We quantified internalization at different time points following a similar but simplified protocol: BTX-biotin internalized via its interaction with K_V_10.1-BBS was detected directly on western blots (Fig. 5B). In the presence of 0.15 µM BTX-biotin at 37°C, HEK cells constitutively internalized 0.9% (±0.4%) of K_V_10.1-BBS surface molecules per minute. This uptake reaction started to show saturation for uptake reactions longer than 45 minutes.

### K_V_10.1-BBS internalization involves several endocytic pathways

**Figure 6:**
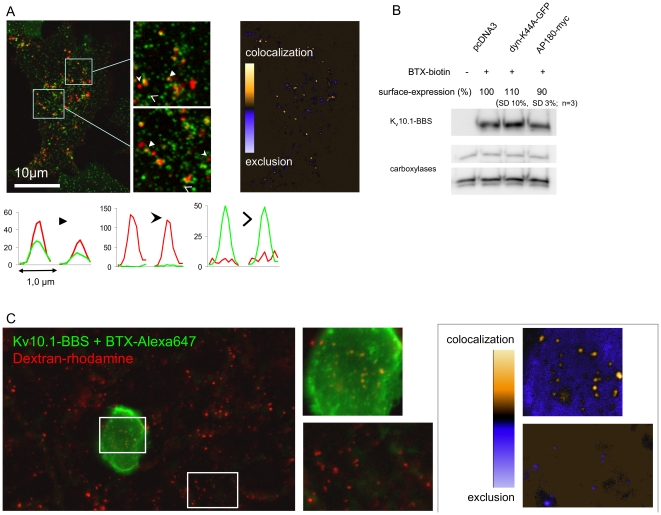
Clathrin-mediated endocytosis is modestly involved in the internalization of K_V_10.1. A) Complexes of K_V_10.1-BBS with BTX-Alexa594 (red) colocalize with clathrin-GFP (green) in punctuate structures (magnified insets, middle) compatible with endosomes. Line profile plots (bottom) through vesicles (white arrows) represent three classes of labeling. The colocalization map (right panel) highlights punctae with a high vs. low degree of colocalization. B) Surface-expression of K_V_10.1-BBS is altered in cells that overexpress components of the clathrin-dependent endocytic machinery. Surface channels were labeled with BTX-biotin, isolated and immune-detected in western blots. Overexpression of the dominant-negative dynamin-K44A led to slight increases in surface expression, while overexpressing AP-180 slightly decreased K_V_10.1-BBS surface-expression. C) K_V_10.1 is internalized by fluid phase uptake. Complexes of K_V_10.1-BBS with BTX-Alexa594 colocalize with dextran-rhodamine, a marker for fluid phase uptake, after 3 minutes of chase reaction. Corresponding ROIs from the merged dual-color image (center) and the intensity correlation image (right) highlight structures with colocalization.

We examined the role of clathrin-mediated endocytosis (CME) for the constitutive uptake described above by testing for colocalization of clathrin-GFP and BTX-Alexa594 upon endocytosis. For this purpose we transiently co-expressed K_V_10.1-BBS and the fusion protein clathrin-GFP [30] and performed pulse chase experiments like described above (Fig. 6A). For analysis, we only considered cells devoid of green fluorescent aggregates. Depending on the position along the cellular z-axis, we could identify a variable number of punctuate signals with green fluorescence, red fluorescence, or both, corresponding to clathrin-GFP and BTX-Alexa594, respectively (Fig. 6A). Intensity correlation analysis (ICA) consistently produced low values for the global Intensity Correlation Quotient (ICQ) of ∼0.2±0.05 (n = 5), indicating dependent staining [48], [49]. Importantly intensity correlation images provided us with 2-D graded maps of colocalization, highlighting objects, i.e. K_V_10.1-BBS containing vesicles, with high degrees of colocalization versus objects with no colocalization. Colocalization was only detectable for a minority of K_V_10.1-BBS containing vesicles (<20%) indicating that CME contributes to K_V_10.1-BBS endocytosis only marginally.

In order to further evaluate the role of CME in K_V_10.1 endocytosis, we co-transfected K_V_10.1-BBS and proteins known to inhibit CME and analyzed K_V_10.1-BBS surface levels in western blots. Co-expression of mutant dynamin, dynamin-K44A-GFP [50], [51], resulted in a slight up-regulation of K_V_10.1-BBS surface levels by 10% (SD = 0.1, n = 3) as compared to cells transfected with empty pcDNA3 vector (data not shown). Analogously, we over-expressed AP180 that can competitively inhibit the formation of clathrin pits [52]. This resulted in a reduction of surface levels by 10% (SD 0.03; n = 3). Based on GFP-expression from pTracer plasmids we observed low transfection efficiencies for these constructs (∼20% of cells) and highly variable levels of protein expression among individual cells.

We also examined whether K_V_10.1-BBS is also internalized by fluid phase uptake, as determined by co-uptake of the fluid-phase uptake-marker rhodamine-dextran (0.1mg/ml) [53] and BTX-Alexa488 (2 µM) in cells expressing either K_V_10.1-BBS or K_V_10.1. After 3 minutes at 37°C we could detect vesicular structures containing rhodamine-dextran, the majority of which also displayed green fluorescence in cells expressing K_V_10.1-BBS (Fig. 6C) but not K_V_10.1 (data not shown). To ascertain that colocalization is specific to cells transfected with K_V_10.1, we produced intensity correlation images. We determined that ROIs containing transfected cells (positive for K_V_10.1 staining) showed more colocalized pixels as compared to ROIs containing untransfected cells (7±4% versus 1±1%, respectively, n = 8, P<0.01). Additionally, we confirmed that no spectral crosstalk occurred from the Alexa-647 to the rhodamine detection channel (data not shown).

The low number of vesicles and weak labeling observed with dextran required detection with epifluorescence illumination, sacrificing optical resolution to sensitivity.

### Internalized K_V_10.1-BBS is rapidly transported to lysosomes

Next we analyzed the role of lysosomal degradation for the K_V_10.1 life cycle. We found that surface-labeled K_V_10.1-BBS is rapidly transported to lysosomes upon internalization.

**Figure 7:**
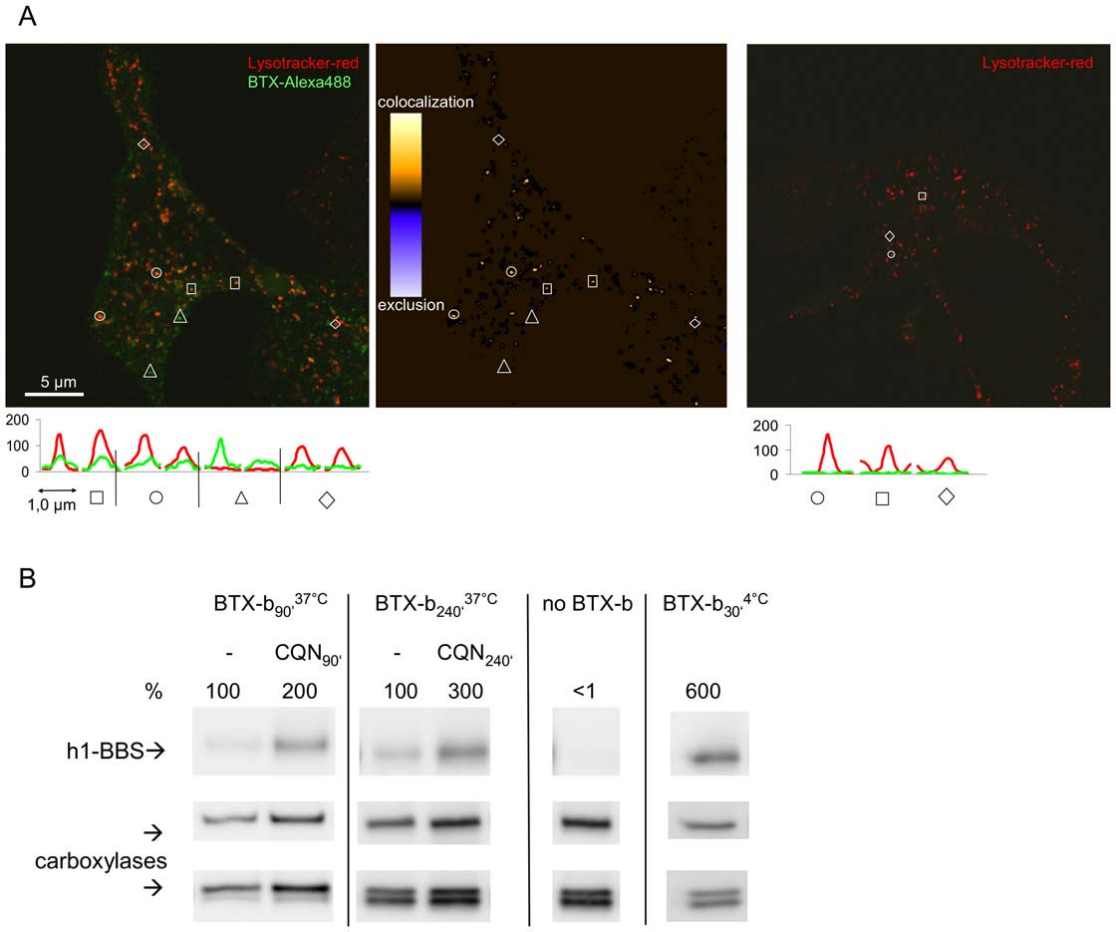
K_V_10.1 is internalized and sorted to lysosomes for degradation. A) Internalized complexes of K_V_10.1-BBS with BTX-Alexa488 (green) colocalize with the lysosome stain ‘lysotracker red’ (red). The presented image was recorded close to the plane of the basal membrane (left). Line profile plots through highlighted punctuate structures distinguish dual- or single-color labeling and shows that lysotracker red did not show signals in the green detection channel (right) and consequently no photoconversion. The intensity correlation image (center) maps punctae with a high degree of colocalization. B) A rescue of internalized K_V_10.1-BBS molecules by the lysosome inhibitor chloroquine (CQN) was detected in western blots (row 1): internalization of K_V_10.1-BBBS complexed to BTX-biotin is shown for ± CQN during 90 minutes in lane 1 & 2 and for 240 minutes in lanes 3 & 4, respectively. For isolation of internalized K_V_10.1-BBS molecules surface labels were removed labeling on ice. Endogenously biotinylated carboxylases were detected to normalize signals for pull-down efficiencies.

HEK cells expressing K_V_10.1-BBS were surface-labeled with BTX-Alexa488 on ice and then incubated at 37°C for 30 minutes. After 20 minutes, Lysotracker-red (LT) was added to the medium. LT is routinely used to identify lysosomes and specifically accumulates in acidified compartments resulting in a red fluorescence stain [54]. We also incubated cells exclusively with LT as a negative control because LT can be photoconverted to green fluorescent dyes under certain circumstances [54]. Object-based analysis of vesicular structures with line profile plots identified objects with colocalizing or exclusively green or red signals, and confirmed the absence of significant LT photoconversion under our experimental conditions (Fig. 7A). ICA images showed some K_V_10.1-BBS positive punctae with strong LT signals (bright yellow), while most punctae displayed very weak or no colocalization with LT (dark yellow to blue). ICA confirmed that individual cells showed a consistent extent of colocalization (ICQ  = 0.19±0.04; n = 5).

In a second step we probed the lysosomal degradation of K_V_10.1-BBS by inhibiting lysosomal acidification in pulse-chase internalization experiments. For this purpose, we applied the lysosomotropic drug chloroquine, a routinely used reagent that inhibits lysosomal acidification and thereby protein degradation while not affecting recycling, as shown for the transferrin receptor [55], [56], Cells expressing K_V_10.1-BBS were incubated in growth medium containing 0.3 µM BTX-biotin in the presence or absence of 200 µM chloroquine for 90 and 240 minutes. After washing off surface-labels at pH3 (Fig. 7B, except for lane 7), cells were lysed and K_V_10.1-BBS BTX complexes were pulled-down like described above. Internalized K_V_10.1-BBS then was detected in western blots. Treatment with chloroquine resulted in an increase in the amount of internalized K_V_10.1-BBS recovered (Fig. 7B). We observed an increase of internalized K_V_10.1-BBS recovered by a factor of 2 for 90 minutes of chloroquine treatment and a factor of 3 for 240 minutes treatment. This amount of internalized labeled channels corresponds to 50% of all labeled surface channels (Fig. 7b) compared to ∼20% without treatment (Fig. 5a).

### A small fraction of K_V_10.1 is recycled to the cell surface

**Figure 8:**
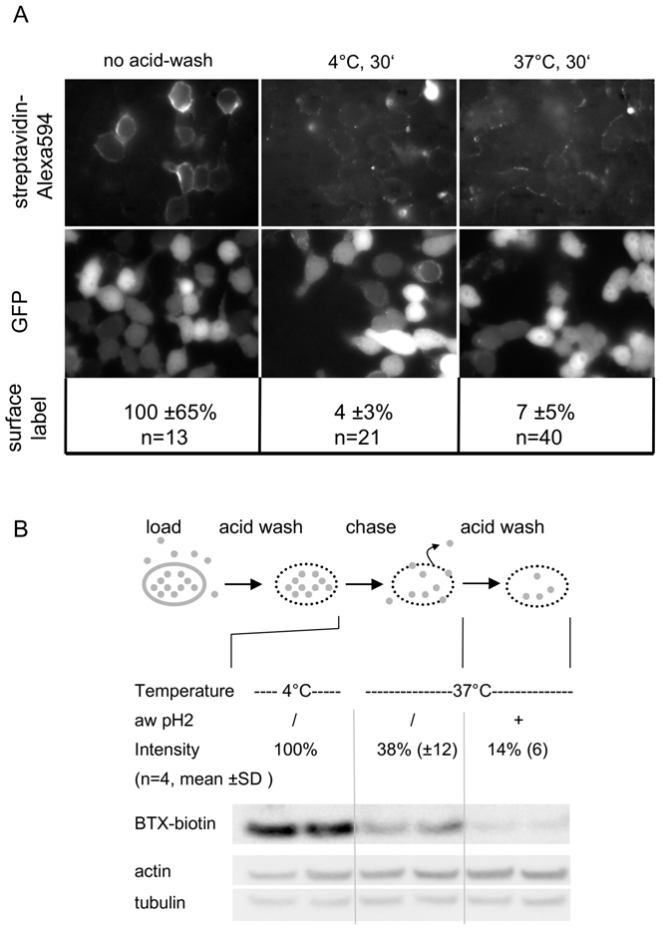
The K_V_10.1 life cycle includes recycling of internalized channels to the plasma membrane. A) Internalized K_V_10.1-BBS channels complexed to BTX-biotin recycle back to the plasma membrane and were detected with streptavidin-Alexa594. Before, BTX-biotin surface-labels had been removed by acid wash. Thereafter incubation at permissive temperatures (30°C) lead to more pronounced membrane signals than at non-permissive temperatures (4°C) (right column versus center, respectively) GFP is expressed from pTracer- K_V_10.1-BBS plasmids as a marker of transfection (second row). Identical exposure times and look up tables were used. Membrane-label intensity was quantified and normalized to membrane-signals before acid wash B) A reduction of intracellular BTX-biotin due to recycling and degradation was detected in western blots (scheme). Intracellular BTX-biotin levels decreased by ∼60% during 30 minutes of incubation at 30°C (lanes 3 & 4) compared to 4°C (lanes 1 and 2). A second acid wash lead to another decrease of BTX-biotin levels by ∼30% presumably by removing recycled BTX-biotin molecules from the cell surface.

To test for a role of recycling in the K_V_10.1 life cycle we directly detected recycled K_V_10.1-BBS molecules at the cell surface (Fig. 8A). In brief, cells stably transfected with K_V_10.1-BBS were loaded with BTX-biotin for 1.5 hours by incubation in medium containing 0.3 µM BTX-biotin. After removing surface-resident BTX-biotin by acid wash cells were put to either 4° or 37°C for 30 minutes. Subsequently, we searched for complexes of K_V_10.1-BBS and BTX-biotin that had recycled back to the cell surface by labeling with streptavidin-Alexa Fluor 594. Fluorescence micrographs consistently showed stronger and more continuous stretches of membrane signal after permissive (37°C) as compared to non-permissive conditions (4°C). We quantified membrane signals from individual cells from different images using ImageJ and detected small but significant (P<0.05) differences in membrane stains under permissive *vs.* non-permissive conditions. Nevertheless membrane signals recorded after incubation at 37°C were only slightly above the intensity range of non-specific background signals. Consequently we set out to detect recycling of K_V_10.1-BBS in a more sensitive analogous biochemical approach.

Instead of labeling with streptavidin-Alexa594 after chase reactions, we applied a second acid wash to remove recycled molecules from the cell-surface before harvesting cells. Comparing the amount of BTX-biotin before and after this second acid wash allowed us to estimate the amount of BTX-biotin that had been recycled to the plasma membrane. We found that approximately 30% of the intracellular BTX-biotin molecules initially internalized during 1.5 hours were recycled back to the plasma membrane within 30 minutes and were therefore accessible to the second acid wash. About 60% of internalized BTX-biotin could not any more be detected after the same time interval, indicating that it had followed a degradation pathway.

## Discussion

We present an analysis of trafficking and transport events that control the intracellular distribution of the oncogenic K^+^ channel K_V_10.1. A very rapid surface turnover that controls the subcellular distribution and life cycle of K_V_10.1channels was observed. We also showed that the endosomal compartment of cancer cells contains significant amounts of K_V_10.1. This finding is relevant for the development of drug-targeting strategies relying on K_V_10.1 [6], [39], [57] In parallel, these new insights and the methods presented might encourage further investigation of the role K_V_10.1 plays in modulating membrane potential during the cell cycle.

The K_V_10.1-BBS construct reported here contains an insertion of the BTX binding site into a very small extracellular loop of a voltage-gated ion-channel (Fig. 1). So far terminal fusions of the BBS to membrane proteins or insertions into large globular domains had been favored as tagging strategies, while K_V_10.1 exhibits only small extracellular loops along with a 83aa pore-forming loop sensitive to manipulation [12], [41], [42], [43], [58], [59], [60]. Tagging K_V_10.1 with this monovalent binding site avoids cross-linking channels as can occur with bivalent antibodies that distort channel structure or affect internalization [61], [62]. Additionally, the small size of the BTX-based affinity labels (∼8.5 kDa) is less likely to disturb transport processes.

Similar electrophysiological phenotypes of wild type K_V_10.1 and K_V_10.1-BBS further support the solidness of this experimental approach. Wild type K_V_10.1 and K_V_10.1-BBS show conserved electrophysiological properties (Fig. 3); this is especially important because it is reasonable to expect trafficking of the channel to be influenced by its activity. Thus, we cannot exclude that the observed shift of the K_V_10.1-BBS half-activation potential to more negative values (Fig. 3B) affects channel trafficking, but one would expect a quantitative rather than qualitative effect on trafficking, since it would represent a change in magnitude otherwise. Importantly, binding of BTX does not substantially affect channel properties either. We therefore conclude that the trafficking properties described here, as long as they depend on the electrophysiological behavior, reproduce well those of the native channel.

Our results confirm that K_V_10.1 shows sparse surface expression as we have described previously for endogenous K_V_10.1 in neurons, and the majority of the channels remain in intracellular compartments (Fig. 2B) [25].

We found that K_V_10.1-BBS is constitutively internalized at a high rate of 0.9±0.4% of K_V_10.1-BBS surface molecules per minute (Fig. 5B). This means that in average a given set of membrane channels is getting internalized once during 105 minutes at 37°C. Constitutive endocytosis commonly involves CME and depends on conserved signals for endosomal and lysosomal targeting, including post-translational modifications such as ubiquitination [35],[63]. Here we provide evidence that CME plays a minor role for K_V_10.1 internalization. A small fraction of K_V_10.1-BBS positive vesicles is clathrin-coated (Fig. 6A). Accordingly overexpression of a dominant mutant version of dynamin, known to inhibit CME, led to slightly increased surface levels of K_V_10.1-BBS. Overexpression of AP-180, reported to block CME [64], slightly decreased K_V_10.1 surface levels. While one would expect that inhibition of CME should result in clearly increased K_V_10.1-BBS surface levels, our results indicate that CME is a minor pathway for endocytosis of K_V_10.1. Certainly redistributions of membrane-associated proteins upon prolonged inhibition of CME can have manifold effects on the equilibrium of two or more pathways of endocytosis, lysosomal sorting and recycling. Contributions of clathrin-independent pathways to internalization events are hard to exclude in general. Interestingly, caveolin is not thought to contribute to constitutive endocytosis but is as well inhibited by the overexpression of mutant dynamin [65]. Actin dynamics were shown to be relevant for internalization in a CME-independent fashion [66], [67]. Here we show that K_V_10.1 internalization clearly is partly due to fluid phase uptake, that is known to be governed by actin-dynamics (Fig. 6B) [68]. Since cargo of fluid-phase uptake is being transported to early endosomes within 5 minutes, we performed this analysis after 3 minutes of chase reaction. Due to the low number of vesicles loaded with dextran and the low intensities observed for rhodamine labeling during this very short pulse, we performed this analysis with epifluorescent illumination, sacrificing optical resolution to sensitivity. Yet, our previous analysis of K_V_10.1-BBS membrane and vesicle stains in confocal sections strongly suggested that structures upon cell loading with BTX-AlexaFluor correspond to internalized vesicles.

Internalized signals started to saturate after 45 minutes of incubation (Fig. 5B) indicating that internalization started to equilibrate with recycling, degradation, or both. Following this line, we established that the high protein turnover observed for K_V_10.1 includes trafficking of K_V_10.1 to lysosomes. We detected high protein turnover in HEK cells expressing the tagged version of K_V_10.1 and rapid transport to lysosomes by colocalization experiments (Fig. 7A). We observed significant rescue of K_V_10.1-BBS by a factor of 2 to 3 upon chloroquine treatment (Fig. 7B), indicating that at least 50% of K_V_10.1-BBS channels internalized during 1 hour undergo lysosomal degradation, provided that, in the presence of chloroquine, K_V_10.1-BBS complexed to BTX-biotin accumulates in lysosomes that show increased pH values and less proteolytic activity. It is unlikely that chloroquine increases the amount of internalized K_V_10.1-BBS by inhibiting the recycling. Chloroquine does not affect the recycling of the transferrin receptor and we did not observe a decrease in K_V_10.1-BBS surface levels as it would be expected for an inhibition of recycling (data not shown) [56].

We also observed that recycling of K_V_10.1 actually occurs at an intermediate rate and is involved in controlling the K_V_10.1 life cycle. Our fluorescent micrographs show faint membrane stains in conditions permissive for recycling compared to signal levels at the beginning of our chase reaction (Fig. 8). Additionally, the membrane labeling is partial and punctuate at several places. We attribute this to a limited access of AlexaFluor-Streptavidin conjugates to membranes between adjacent cells and to the extensive cross-linking that tetra-valent untitrated streptavidin can induce. According to our biochemical analysis ∼30% of intracellular BTX-biotin molecules are recycled back to the surface within 30 minutes. It is important to note that the interaction of BTX-conjugates and K_V_10.1-BBS survived reduced pH values, which is relevant in order to maintain labeling inside acidified endosomes of ∼pH6 (Fig. 5A). Based on the uptake and recycling rates measured we can estimate that 15 to 25% of K_V_10.1-BBS surface channels are recycled within 30 minutes. The outcome of our microscopic analysis of recycling clearly favors the lower estimate. In any case, a significant fraction of endocytosed K_V_10.1-BBS is being sorted to be recycled and consequently presents an intracellular store of K_V_10.1.

We can only speculate about the physiological significance of the degradation and recycling described. The most likely explanation is that the observed constitutive internalization, degradation and recycling result from signals for the endocytic machinery that originally have evolved to regulate K_V_10.1 surface expression in neurons. Probably surface expression is tightly regulated in both cancer cells and neurons by a similar conserved mechanism. Consequently, overexpression of K_V_10.1-BBS in neurons presents the most straightforward way to gain insights into these questions. With respect to the fluid-phase uptake of K_V_10.1-BBS detected, it is tempting to ask whether interactions of K_V_10.1 with the filament system might modulate channel activity and localization [69], [70], along with surface-expression. In return, K_V_10.1 might simultaneously disturb actin dynamics upon ectopic overexpression. This idea is particularly interesting with respect to the debated mechanisms of how K_V_10.1 promotes cancer. Analysis of actin-filament dynamics upon overexpression of K_V_10.1-BBS might shed light on this question [71].

### Conclusion

We present a tagged version of the ‘ether-á-go-go’ ion channel K_V_10.1 where the α-bungarotoxin-binding site has been inserted within an extracellular loop. Using this tagged channel named K_V_10.1-BBS we show that K_V_10.1 is rapidly internalized in a constitutive manner in HEK cells (at 0.9%/minute). Our data also shows that the high K_V_10.1 protein turnover involves surface-expression followed by constitutive internalization and degradation in lysosomes (≥50% of internalized channels per hour). In parallel, internalized K_V_10.1 is also being recycled back to the plasma membrane (∼15% of membrane molecules in 30 minutes). This high surface turnover rate and involved mechanisms might be crucial mechanisms that evolved in order to maintain channel targeting in neurons and are likely to contribute to the oncogenicity of K_V_10.1.

## Methods

### Chemicals

D7163 dextran, Rhodamine Green3000 MW and all α-bungarotoxin conjugates were purchased from Invitrogen. Other chemicals were from Sigma-Aldrich.

### Plasmids, cRNA and transfection

The α-bungarotoxin-binding site (BBS) was inserted into K_V_10.1 at aa position 318. To do this, an *Age*I restriction site was inserted at ORF position 952 to 957 into the cDNA using the QuickChange XL site-directed mutagenesis kit (Stratagene). A short DNA-fragment encoding the BBS and short flanking linker sequences was produced by annealing complementary custom-synthesized oligonucleotides (Metabion). This DNA-fragment was cloned into the *Age*I restriction site resulting in a 27–residue insert: TGGSSG*WRYYESSLEPYPD*GSGSTG, including the minimized α-bungarotoxin-binding site (italics) [43]. This K_V_10.1-BBS construct was established in parallel in vectors based in pTracer and pcDNA3 and also in a K_V_10.1 fusion to monomeric Venus in pcDNA3, all of which have been described elsewhere [37], [72], [73]. Transfections were performed with Lipofectamine 2000 (Stratagene) according to the manufacturers' instructions. cRNA was produced with the T7 mMessage mMachine kit (Ambion) from our constructs in pcDNA3 after linearization of plasmids with *Nae*I. Oocyte preparation and electrophysiological recordings were performed as described elsewhere [74].

### Cell Culture

HEK293 cells (DSMZ, Germany) stably transfected with pTracer- K_V_10.1-BBS and pTracer- K_V_10.1 were selected and maintained using Zeozin (Calya, 3 mg/ml in culture medium).

### Isolation of K_V_10.1-BBS and detection in western blots

Surface expressed K_V_10.1-BBS channels were labeled with 0.15 µM or 0.3 µM α-bungarotoxin-biotin in PBS supplemented with 0.1% BSA or in culture medium at the indicated temperatures. After two washing steps, cells were harvested and lysed in buffer (1% Triton X-100, 150mM NaCl, 5mM EDTA, 5mM KCl plus Roche Complete protease inhibitor cocktail) for 20 minutes. Following drug treatment, cells were lysed in buffer containing 10% 1-,4-dioxane to reduce non-specific protein precipitation. Streptavidin-coated magnetic beads (Dynabeads type T1; Invitrogen) were added to equalized amounts of pre-cleared lysates (protein concentration was determined by BCA-assay, Pierce) to bind α-bungarotoxin-biotin complexed to solubilized K_V_10.1-BBS for 30 minutes on ice. After washing 3 times with lysis buffer and once with TBS, retained proteins were eluted from beads with LDS sample loading buffer (Invitrogen) and analyzed by standard SDS-page and immunoblotting. Membranes were blocked with 1% casein in TBST (0.1% Tween-20). K_V_10.1 was detected after consecutive incubation of membranes with a polyclonal anti- K_V_10.1 antibody [31].

Alternatively α-bungarotoxin-biotin and endogenous biotinylated carboxylases were directly detected after SDS-page and western blotting with streptavidin-peroxidase (Invitrogen). To exclusively detect internalized α-bungarotoxin-biotin, adherent cells were subjected to acid wash at pH 3 for 2 minutes at 4°C prior to harvest. As reference, actin and tubulin were detected using the corresponding antibodies. Blots were developed using Millipore Immobilon system and signals detected in a BioRad ChemDoc luminescence detection system. Luminescence signals were quantified by densitometry using the ImageJ ‘Gel Analyzer’ function. Where necessary, collected signals were normalized to signals from biotinylated endogenous carboxylases and/or actin.

### Two-electrode voltage-clamp recordings

We performed two-electrode voltage-clamp recordings to measure current-voltage relationships and channel activation kinetics as described previously [45].

Briefly, recordings were performed one day after cRNA injection, using a Turbo TEC-10CD amplifier (NPI electronics) at room temperature. The intracellular electrodes had typical resistance of ∼1.5 MΩ when filled with 2M KCl. The extracellular measuring solution contained 115 mM NaCl, 2.5 mM KCl, 1.8 mM CaCl_2_, 10 mM HEPES/NaOH, pH 7.2.

Data acquisition and analysis were performed with the Pulse-PulseFit (HEKA Electronics) and IgorPro (WaveMetrics) software packages. Current records were sampled at 10 or 20 kHz and filtered at 1 kHz. The cells were held at −100 mV membrane potential. The applied voltage protocols are described in the figure legends. No leak current subtraction was carried out.

Voltage dependence of activation was estimated by fitting the mean normalized current-voltage relationships measured according to a Hodgkin-Huxley formalism taking also into account the rectification at positive potentials (Eq 1) [75]:



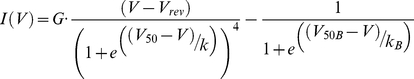
(1)where G is the total conductance, V_rev_ the reversal potential (fixed at the value of −98.5 mV according to Ju and Wray 2002, V_50_ the potential of half activation per subunit and *k* the slope factor; similarly the parameters V_50B_ and k_B_ characterize the rectification at positive potentials [76].

### Patch clamp recordings

Patch clamp recordings were performed on HEK293 cells stably transfected with pTracer- K_V_10.1-BBS. Currents were recorded before and after incubation of the cells with 1 µM α-bungarotoxin-Alexa594 for 10 minutes at 4°C followed by two washing steps to measure current densities in the whole cell configuration of the patch clamp method [77] using an EPC9 amplifier and Pulse software (HEKA). Currents were filtered at 10 kHz and digitized at 50 kHz. Patch pipettes were pulled from Corning #0010 glass (World Precision Instruments) to resistances of 1–2 MΩ. Solutions contained in mM: Internal: 100 KCl, 45 N-methyl-D-glucamine, 5 1,1-bis(*O*-aminophenoxy)ethane-N,N,N′,N′-tetraacetic acid (BAPTA), 5 EGTA, 1 MgCl_2_, 10 HEPES pH7.4; External: 160 NaCl, 2.5 KCl, 2 CaCl_2_, 1 MgCl_2_, 8 Glucose, 10 HEPES, pH 7.4. We used the automated capacity compensation feature of the amplifier to estimate cell capacity and series resistance, which was compensated to 85%.

### Epifluorescence and confocal imaging

Surface K_V_10.1-BBS channels were labeled with fluorescent (coupled to Alexa Fluor 488, Alexa Fluor 594 or Alexa Fluor 647 –Invitrogen-) α-bungarotoxin conjugates at either 1 µM in saline buffer (140 mM NaCl, 4.5 mM KCl, 2 mM CaCl_2_, 1 mM MgCl_2_, 10 mM glucose, 10 mM HEPES pH 7,4, 0.1% BSA) for 10 minutes on ice or at 0.3 µM in culture medium at 30°C for the indicated time intervals. To remove excess label, cells were washed in saline buffer 3 times. Alternatively, surface-channels were labeled detecting α-bungarotoxin-biotin bound to K_V_10.1-BBS channels (compare above) with 0.3 µM streptavidin-Alexa Fluor 594 conjugates in PBS for 15 minutes on ice.

Lysosomes in living cells were labeled with 100nM Lysotracker red (Invitrogen) at 30°C in medium for 10 to 20 minutes immediately before imaging. The plasma membrane was stained with 3 µg/ml FM 4–64 immediately before imaging. Clathrin-GFP was co-expressed transiently in HEK293 cells for 24 h before imaging.

Wide-field image acquisition was performed on an inverted Axiovert 200M (Zeiss) microscope equipped with 40x and 63x oil-immersion objectives and a Hamamatsu Orca12 camera, using standard filter-sets for Alexa Fluor 488, Alexa Fluor 546 and Alexa Fluor 633.

Confocal imaging was performed on a Leica SP2 confocal microscope with a 40x (NA 1.3) or 63x (NA 1.4) oil immersion objectives imaging at pixel sizes of 70x70nm to 150x150nm. Live cell imaging was performed in saline buffer (140 mM NaCl, 4.5 mM KCl, 2 mM CaCl_2_, 1 mM MgCl_2_, 10 mM glucose, 10 mM HEPES pH 7,4, 0.1% BSA) at either room temperature or 30 to 37°C within a live-cell chamber. The indicated settings (exc./em.) were applied for imaging combinations of GFP/Alexa Fluor 594 (488nm/505–560nm; 594nm/610–680nm, pinhole at 1AU, 63x), Alexa Fluor 488/Lysotracker-Red (488nm/500–530nm and 575–700nm, pinhole at 1.25AU, 40x), Alexa Fluor 488/FM4–64 (488nm/500–535nm and 555–620nm, pinhole at 0.75 AU, 40x, sections at a distance of d = 0.3 µm), respectively.

Colocalization analysis was performed based on line profiles through punctuate signals with 3 pixels width and intensity correlation analysis (ICA) with imageJ. ICA tests whether intensities in two channels vary in parallel, independently or in a segregated manner, corresponding to colocalization, random distribution or exclusion of red and green signals, respectively [48], [49]. Like Pearson's and Manders' coefficients, ICA produces a statistical parameter of global colocalization, called Intensity-Correlation-Quotient (ICQ), which is helpful to summarize colocalization from several dual-color images. As all other global colocalization procedures, the ICQ is also sensitive to threshold selection and ICQ values do not directly correspond to object-based colocalization. Yet, as a major additional benefit, ICA produces a two-dimensional map of graded colocalization, where positive pixel-values correspond to a high degree of colocalization (here maximum values shown in yellow), zero values to random distribution (here: black) and negative values to mutual exclusion of labels (here minimum values shown in blue). This allowed us to identify objects with high degrees of colocalization. We performed our ICA analysis after background subtraction in both fluorescent detection channels and restricted it to the image segment corresponding to the top 60% of pixel values in the K_V_10.1-BBS channel.
